# Percutaneous fixation of posterior pelvic ring lesions in the elderly: current evidence and recent advances

**DOI:** 10.1007/s00068-026-03231-3

**Published:** 2026-07-01

**Authors:** Emmanouil Liodakis, Sebastian Schreiber, Vassilis P. Giannoudis, Tobias Fritz, Peter V. Giannoudis

**Affiliations:** 1 Department for Trauma, Hand and Reconstructive Surgery, Saarland University, Homburg, Germany; 2 Academic Department of Trauma & Orthopaedics, Leeds Teaching Hospitals, Leeds, University of Leeds, Leeds, UK

**Keywords:** Posterior pelvic ring, Fragility fractures, Percutaneous stabilization of the pelvis, Sacroiliac, Transsacral, Sacronail, Photodynamic, Scoping review

## Abstract

**Introduction:**

Percutaneous fixation techniques are increasingly used in the management of posterior pelvic ring lesions in the elderly. As the incidence of fragility fractures continues to rise, a growing number of implants and surgical techniques have been developed to address the specific challenges of this vulnerable population. The goal of this scoping review is to address the following questions: 1) What are the latest advances in percutaneous fixation of posterior pelvic ring fractures? 2) What are the clinical results and current level of evidence supporting these techniques?

**Methods:**

A systematic search using the keywords “((Percutaneous) OR (Minimal* invasive) OR (Transsacral) OR (Innovative implant)) AND ((Pelvic fragility fracture) OR (Sacral fracture))” was performed in PubMed and Cochrane Library using PRISMA-ScR guidelines. Studies published from the 11^th^ of August 2020 up to the 11^th^ of August 2025 were included and assessed independently by two reviewers.

**Results:**

A total of 334 studies were found, of which 107 were included in this review following the screening process. Of these, 22 studies pursued a biomechanical approach. Various new implants (e.g. ISG-Rod System, SI-Plate, TriFix, SACRONAIL®) and technologies (e.g. 3D computer-assisted navigation/O-arm/Robot) are described in the recent literature. Using navigation significantly reduces the rate of screw malposition. Additional cement augmentation can reduce the length of a patient’s stay in hospital. (Bilateral) transsacral fixation reduces the risk of fracture progression. Novel implants, such as curved implants and photodynamic nails, offer promising alternatives for patients with dysmorphic sacra.

**Conclusion:**

Iliosacral screw fixation is considered the gold standard for the percutaneous stabilization of the posterior pelvic ring. However, given the vast range of new implants and technologies available, decisions should always be made on a case-by-case basis. High-quality evidence studies comparing new implants with conventional SI screws are desirable.

## Highlights

Transsacral percutaneous fixation is increasingly seen as a safe, effective minimally invasive treatment for geriatric posterior ring fractures

The use of Navigation appears a safe and efficient method for percutaneous screw placement

Multiple new implants have been described for management of these injuries.

Level I evidence is still lacking for treatment options in the management of posterior pelvic ring lesions with percutaneous fixation

## Introduction

Due to demographic changes and the increasing prevalence of osteoporosis, the incidence of pelvic fragility fractures is steadily rising. This trend has been described as a “new silent epidemic” [1].

Pelvic ring injuries in the geriatric population require careful consideration and appreciation of the fracture morphology. These fractures can be classified according to the comprehensive classification of fragility fractures of the pelvic ring (FFP) [2], the OF (osteoporotic fractures) pelvis classification [3] or the Tile classification [4]. The posterior ring osseo-ligamentous structures provide 85% of intrinsic stability within the pelvic ring [5]. Reduction and fixation of this is therefore of paramount importance. Several centres have reported positive results in the fixation of posterior ring alone in Tile B/Tile C fractures compared to dual anterior and posterior fixation. Findings reported were lower blood loss, fewer post-operative complications and better radiological outcomes [6].

In cases where operative treatment is warranted, percutaneous iliosacral screw fixation is considered the gold standard, as supported by several studies reviewing the current evidence [7–21]. This preference is based on two key considerations: Firstly, geriatric patients are frail and cannot tolerate substantial blood loss or prolonged operative procedures; and secondly, open approaches to the posterior pelvic ring carry a high risk of morbidity and mortality, such as wound complications and postoperative infections [22].

Despite their advantages, percutaneous techniques present several challenges. The available bony corridors in the pelvis are often narrow and curved, particularly in cases of sacral dysmorphism, which is present in approximately 40% of individuals [23]. Achieving closed reduction in displaced fractures can be difficult due to limited visualization and the challenges of manipulating fracture fragments. If the initial placement of the drill or guidewire is suboptimal, redirecting it is technically demanding. Moreover, poor bone quality in elderly patients increases the risk of screw loosening following percutaneous fixation [24]. Finally, as in all pelvic procedures, critical anatomical structures are at risk, yet cannot be visualized during percutaneous fixation. These include, for example, the spinal canal, the L5 nerve root and the iliac vessels.

In light of the aforementioned challenges, a range of novel techniques and implants have been introduced in recent years to improve the percutaneous management of pelvic fragility fractures.

The aim of this scoping review was to answer the following questions:

1. What are the latest advances in percutaneous fixation of posterior pelvic ring fractures in the elderly?
2. What is the current level of evidence supporting these techniques?

## Materials and methods

### Search strategy

**Fig. 1:**
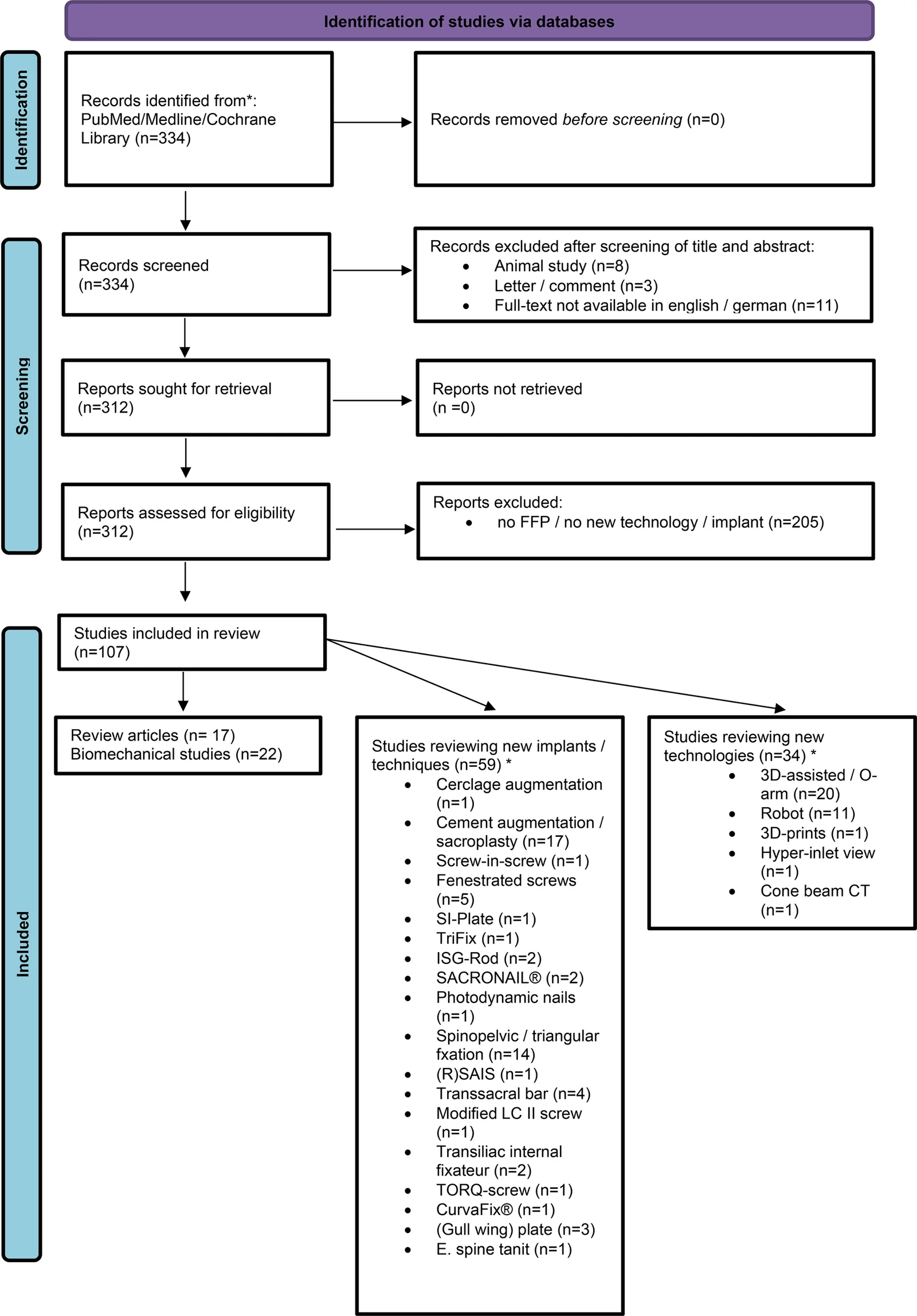
The PRISMA flowchart summarizes the search strategy used. * Please note: some studies fit into more than one category, which explains why there are duplicate listings

A systematic search according to PRISMA-ScR [25, 26] using the search terms “((Percutaneous) OR (Minimal* invasive) OR (Transsacral) OR (Innovative implant)) AND ((Pelvic fragility fracture) OR (Sacral fracture))” in PubMed and Cochrane Library, was performed by the first (L.E.) and second author (S.S.). The search was limited to the languages English and German. The search strategy is outlined in Fig. 1.

### Eligibility criteria

Studies were included if they met the following criteria:

1. Studies investigated the new implants and technologies in relation to pelvic fragility fractures;
2. The outcomes included clinical and/or biomechanical and/or radiological data;
3. Clinical studies with the full-text paper published within the last 5 years (from the 11^th^ of August 2020 to the 11^st^ of August 2025).

### Exclusion criteria

Studies were excluded if they met the following criteria:

1. Animal studies, conference abstracts, letters, or comments;
2. Full-text paper written in English or German was not available.

## Results

A total of 334 records were identified using the predefined search terms. After title and abstract screening by two reviewers (first and second authors), 107 studies were deemed relevant to our research questions. Noteworthy, 16 studies focused on minimally invasive spinopelvic fixation [27–42].

Their key findings are summarized in Tables 1, 2, 3.

**Table 1:** Innovative implants/surgical techniques used to stabilize the posterior ring in fragility fractures (* “0” indicates a review article)

New Implants	Studies (Author, Journal, Year) (number of included patients/artifi-cial pelvises/cadaver)*	Total number of patients (artificial pelvises/cadaver)*	Characteristics	Number of clinical studies in the last 5 years	Highest Level of evi-dence	Compara-tive studies with SI screws	Advantages	Disadvantages/Compli-cations/Revisions	Outcomes	Biomecha-nical studies
**Cerclage augmen-tation**	**Berk** et al., 2023 (24)	0 (24)	Cannulated S1 and S2 transsacral screws combined with cable or wire cerclage augment-ations	0 (1 biomecha-nical)	V	**Berk** et al., 2023	Loosening of the screws is practically impossible, as long as there is no failure of the cerclage	Necessity for an additional contralateral incision that will be required to insert the cable or wire into the S2 screw, patient discomfort	-	**Berk** et al., 2023
**Cement augmen-tation/Sacro-plasty**	**Graul** et al., 2025 (5); **Maldinez** et al., 2025 (49); **Gewiess** et al., 2024 (0); **Nasralla** et al., 2024 (11); **Singh** et al., 2024 (0); **Sayed** et al., 2024 (1); **Ganeshan** et al., 2023 (1); **Lodde** et al., 2023 (1); **Caudron** et al., 2023 (1); **Haveman** et al., 2022 (59); **Boeken** et al., 2022 (12); **Sag** et al., 2022 (12); **Lotan** et al., 2022 (8); **Lodde** et al., 2021 (21); **Kao** et al., 2021 (44); **Lee** et al., 2020 (40); **Hartensuer** et al., 2020 (448)	647 (26)	Augment host bone density by inserting bone cement at the tip of the screw (before inserting the screw), Sacroplasty: transiliac, long-axis, “Eiffel-Tower technique”, “XX-technique”, short-axis, co-axial approach; Typically, 1.5–7.5 mL of polymethyl methacrylate is injected into the affected sacral ala	15 (+2 biomecha-nical)	II	0	Pullout strength was significantly greater; Co-axial sequential injection sacroplasty potentially even more safety over conventional sacroplasty techniques; regardless of the approach, sacroplasty offers good short-term and long-term clinical outcomes with minimal complications if performed properly, has been postulated to create an improved biomechanical environment for fracture healing	With cyclic loading, the overall construct failed at a similar rate with the failure occurring at the iliac wing instead, possible leakage of cement into the fracture gap/spinal canal and cement embolism, sacroplasty: close relationship between the cement injection and the sacral foramina can make this technique difficult; spinal/foraminal extravasation	Triangular stabilization using two obliques and an additional transiliosacral screw: Cement augmentation did not provide any significant gain in maximum force ; sacroplasty: significant pain reduction and improvements in functional mobility and quality of life, higher patient satisfaction	**Graul** et al., 2025; **Lodde** et al., 2021
**Screw-in-screw**	**Zderic** et al., 2021 (27)	0 (27)	standard 7.3-mm cannulated SI screw, adopted to accommodate proximally a 2.7-mm locking head screw at a 15° set angle. An additional threaded hole in the SI screw head is implement-ted to interlock the 2.7-mm locking screw	0 (1 biomecha-nical)	V	0	Locking principle renders the implant additionally angular-stable, and hence contributes to an overall enhanced construct stability, requires minimally more effort compared to standard SI screw fixation	Screw-in-screw fixation did not provide same stability as transsacral fixation	-	**Zderic** et al., 2021
**Fenes-trated screws**	**Lodde** et al., 2023 (1); **Gewiess** et al., 2023 (1); **Haveman** et al., 2022 (59); **Kress** et al., 2022 (28); **Suero** et al., 2021 (10)	89 (10)	Augment host bone density by inserting bone cement at the tip of the screw	4 (1 biomecha-nical)	III	0	Offers potential benefits for patients with osteoporotic bone, improved stability over a single non-augmented screw, significant increase in fixation strength, reduce screw loosening	Spinal/foraminal extravasation, embolic episodes or neurologic symptoms	Successful treatment of FFP IV fracture	**Suero** et al., 2021
**SI-Plate, Silony Medical GmbH**	**Grüneweller** et al., 2023 (16)	0 (16)	Iliosacral screw with a pre-mounted plate, allowed the placement of an additional short angular stable screw in the ilium	0 (1 biomecha-nical)	V	0	The double thread design is used to allow rapid insertion combined with good primary stability through some interfragmentary compression	Stability significantly lower compared to TriFix	The SI-Plate and TriFix showed comparable stiffness values. The values for fracture gap angle and screw tip cutout were significantly lower for the TriFix compared to the SI-Plate. In addition, the number of cycles to failure was significantly higher for the TriFix	**Grüneweller** et al., 2023
**TriFix system, Silony Medical GmbH**	**Grüneweller** et al., 2023 (16)	0 (16)	Fenestrated iliac screw and an iliosacral screw with a pre-mounted washer. The iliosacral screw is inserted through the “fenestra” of the ilium screw by using an aiming arm device	0 (1 biomecha-nical)	V	0	Quasi-angular stable fixation is provided. The TriFix design allows stepwise and modular surgical treatment of the dorsal, pelvic ring according to the biomechanical needs of the fracture or instability. The primary stability of the construct is increased by the quasi-angle stable connection of the iliac screw and the iliosacral screw in combination with the additional medial support of the iliosacral screw. As mentioned above, the modular design allows for easy extension to spinopelvine stabilization	The values for fracture gap angle and screw tip cutout were significantly lower for the TriFix compared to the SI-Plate.	The SI-Plate and TriFix showed comparable stiffness values. The values for fracture gap angle and screw tip cutout were significantly lower for the TriFix compared to the SI-Plate. In addition, the number of cycles to failure was significantly higher for the TriFix	**Grüneweller** et al., 2023
**ISG-Rod/** **screw-rod fixation**	**Fischer** et al., 2024 (106); **Gamada** et al., 2022 (6)	202 (0)	Cannulated and counterable implant	2	III	**Fischer** et al., 2024	Advantages with regard to the duration of surgery and fluoroscopy compared to sacral bar and SI screw, may be suitable for FFP IVb fractures	Risk of pain and irritation at the screw insertion site	The risk of incarceration of sacral nerve roots due to the applied compression appears negligible, as the effect is most likely to occur in the bone-reduced alar zone. Relevant implant malpositions could not be detected	0
**Sacronail**	**Unthan** et al., 2024 (3); **Marintschev** et al., 2023 (27)	30 (0)	Minimally invasive bilateral fixed angle locking system, constant diameter of 8 mm and is available in lengths between 135 and 194 mm. The fixed angle of the locking screws to the nail axis is 70 degrees, and represents the anatomical corridor of the ilium axis, which was previously determined on CT-data specimens	2	V	0	Allows immediate full weight bearing, risk of secondary surgical complications is low. Improvement in pain scores and mobility, very rigid, bilaterally fixated construct in the ilium	Preliminary, the advantages and limitations of the new implant compared to standard operative procedures must be critically evaluated in further randomized prospective studies	Fixation of the posterior pelvic ring with immediate full weight bearing. No wound healing or infectious complications were observed up to now. The two neurological adverse events following surgery and postoperative mobilization were not associated with malreduction or implant misplacement	0
**Photodynamic nails (PDN)**	**Clunk** et al., 2025 (14)	(14)	Minimally invasive percutan-eous technology consisting of patient conforming, light-sensitive polymer implants	1	III	0	Low-risk procedure, offers swift pain relief and significant improvements in patient function, customizable nature of PDNs allows for adaptation to individual pelvic anatomy and potentially reduces the risk of mechanical failure	Thromboembolism (1 patient), very little data available so far	safe surgical technique that effectively restores patient ambulatory function and provides rapid pain relief	0
**Spino-pelvic/tri-angular fixation**	**Gewiess** et al., 2025 (88)**; Graul** et al., 2025 (5); **Girgis** et al., 2025 (0); **Zhao** et al., (2025) (1)**; Voort** et al., 2024 (5); **Saiz** et al., 2024 (82); **Sevillano-Perez** et al., 2024 (20)**; Ganeshan** et al., 2023 (1); **Mendel** et al., 2023 (34); **Mendel** et al., 2021 (61); **Yoshimura** et al., 2021 (22); **Riesner** et al., 2021 (23); **Spalteholz** et al., 2020 (20); **Obid** et al., 2020 (13)	349 (26)	Consists of pedicle screw fixation into 1 or 2 lumbar spine levels in conjunction with iliac bolts and pelvic fixation with iliosacral or transsacral–transiliac (TSTI) screws depending on the available first (S1) or second (S2) sacral segment osseous fixation pathways	10 (+4 biomecha-nical)	II	**Gewiess** et al., 2025; **Mendel** et al., 2023	Many variations/constructs possible: biomechanical superiority of a modified triangular osteosynthesis construct using S1 pedicle screws, iliac bolts, and transsacral–transiliac screws (L5–S1-IB + TSTI); reducing low back pain and restoring mobility; A triangular-shaped fixation technique for the sacrum results in a comparable stability to that with cement augmentation of the screws, without the risk of cement leakage; modern MIS approaches available resulting in faster recovery, reduced blood loss, and fewer complication	Pedicle screw mispositioning reportedly necessitates revision rates ranging from 0.3% to 8.6%; pedicle perforation poses risks to the intervertebral disc, nerve roots, spinal cord, and paraspinal muscles	Compared to bisegmental transsacral stabilization: no difference in blood loss, fluoroscopy time. In-patient mortality was low; in SP: mobility was significantly lower than before complaints; significant outcome improvement and sufficient fracture healing in SP	**Graul** et al., 2025; **Zhao** et al., 2025; **Voort** et al., 2024; **Sevillano-Perez** et al., 2024
**RSAIS**	**Ma** et al., 2025 5 (15)	5 (15)	Percutan-eous retrograde SAIS (RSAIS) to overcome as spinous processes can block percutan-eous implantation of SAIS	0 (1 bio-mechanical)	IV	0	RSAIS fixation in low bone density offers superior stability compared to SIS and TSTIS; is simple, safe, and accurate, providing satisfactory fixation effects	The cross-sectional area of the RSAIS path (about 15 mm) is smaller, with only one Φ 6.5–8.0 mm screw allowed to implant in one spinal segment; the sacroiliac joint dislocation and peri‑sacroiliac joint longitudinal fracture with unsatisfactory reduction is not applicable; the direction of the screw trajectory is not perpendicular to the joint surface, and the screw does not have the same compression and convergence effects for sacroiliac joint dislocation as the classic SIS	-	**Ma** et al., 2025
**Trans-sacral bar**	**Regenbogen** et al., 2024 (21); **Mendel** et al., 2024 (73); **Rommens** et al., 2022 (64); **Wagner** et al., 2021 (85)	243 (0)	Transsacral bar with washers and nuts placed on both sides reduces the risk of implant loosening	4	II	0	Provides more stability than bidirectional IS screws	In case of a dysmorphic sacrum it can be impossible to fit a transverse bar into the S1 corridor; implant loosening very rare; opening the soft tissues in order to cut the bar at the right length could be a critical maneuver	The SF-8 Physical and Mental Component Scores, Parker Mobility Score and Numeric Rating Scale showed moderate values comparable to those of the same age group without FFP	0
**Modified LC II screw**	**Deng** et al., 2024 (62)	62 (0)	Modified LC-II screw placement method involving sacroiliac joint fixation, resulting in 3-layer cortical fixation; entry point is more lateral and enters the sacroiliac joint through the iliac osseous corridor at an angle of 5°–10° to the osseous corridor of the LC-II screw	1	III	0	Higher fixation stability and facilitates early mobility; reducing the risk of the in–out-in phenomenon	Lack of a specific fluoroscopic position; risk of cutting the medial cortex of the iliac bone; risk of collateral damage resulting from screw penetration	Significantly shorter operative time compared to conventional LC-II, no significant difference in Majeed Score	0
**Trans-iliac internal fixateur**	**Shaalan** et al., 2024 (51); **Kassem** et al., 2023 (72)	123 (0)	Treatment of sacral fractures without spinopelvic dissociation: pedicle screws in both posterior iliac crests, combined with a transverse rod crossing the midline of the posterior sacrum	2	II	**Shaalan** et al., 2024	Better reduction control, reduces the risk of neurological complications, and can be performed without the need for intraoperative images; low blood loss, low complication rates, and high union rates	Longer incision length	No statistically significant differences: final clinical assessment, radiological assessment, the need for another operation, complication rates, the time of union, intraoperative blood loss	0
**TORQ-screw (iFuse-TORQ, SI-BONE, Santa Clara, CA)**	**Ganeshan** et al., 2023 (1)	1 (0)	Implants designed to achieve fracture stabilization, SI joint fixation, and fusion	1	IV	0	3D-printed titanium surface, similar to cancellous bone		Sacral kyphoplasty + percutaneous osteosynthesis and SI joint fusion = effective in means of stabilizing a U-type fracture with prior lumbopelvic fixation, relieving pain, and improving mobility both acutely and long term	0
**Curvafix** **(Curva-Fix, Inc., Bellevue, WA, USA)**	**Miller** et al., 2022 (1)	1 (0)	Novel intramedullary implant used to treat a patient with a pelvic fragility fracture	1	IV	0	Can be redirected along the irregular bony curvature of the pelvis, easier to implant in certain situations, including anterior ring corridors, dysmorphic S1 corridors, Once the implant has a curved path and is locked, it is nearly impossible to back out	-	Allows for timely, minimally invasive surgical stabilization of pelvic fractures, allowing for immediate weight bearing and decreased pain in elderly patients	0
**Gull wing plate (GWP; OMIC Corporation, Shiga, Japan); Minimaly invasive posterior locking compression plate (MIPLCP)**	**Mitsuzawa et al., 2022 (3); Schmerwitz** et al., 2021 (1); **Schmerwitz** et al., 2021 (2) (53)	57 (0)	GWP: re-contoured anatomical locking plate with two cancellous screws and four locking screws, MIPLCP: standard LCP	3	III	0	GWP: simple and safe procedure with fewer complications and less radiation exposure than percutaneous screw fixation	Contraindications for GWP: significantly displaced fractures (especially in the cranial/caudal direction) and bilateral severe injuries around the sacroiliac joints; severely damaged soft tissue, such as a Morel–Lavallée lesion; Risks: intraforaminal screw placement, injury to the sacral foramen	Immediate full weight bearing permitted, relatively low complication rate, low radiation and moderate operation time and a good functional outcome	0
**E. spine tanit (Euros, France)**	**Sawada** et al., 2022 (1)	1 (0)	Novel posterior within ring fixation technique using a combination of iliac screws and an implant that locks the original iliosacral screw in the sacrum	1	IV	0	Small skin incision, IS screw can be locked in the connector, torsional position, no intraoperative navigation is required	Cannot be applied in cases, in which there is vertical shear dislocation, difficult to control the depth of the sacral connector	Patient started walking unaided 2 weeks after the surgery, suggesting a good outcome of this surgical approach to FFP	0

**Table 2:** Innovative technologies used to assist precise intraoperative implant placement in the posterior pelvic ring in fragility fractures (* “0” indicates a review article)

New technologies	Studies (Author, Journal, Year) (number of included patients/artificial pelvises/cadaver)*	Total number of patients (artificial pelvises/cadaver)*	Characteristics	Number of clinical studies in the last 5 years	Highest Level of evidence	Comparative studies with conventional SI screws	Advantages	Disadvantages/Complications/Revisions
**3D computer-assisted navigation/O-arm**	**Gilli** et al., 2025 (158); **Sola** et al., 2025 (36) **; Klingebiel** et al., 2025 (208); **Mizutani** et al., 2025 (72); **Mantilla-Mayans** et al., 2025 (53); **Haveman** et al., 2025 (0); **Link** et al., 2025 (141); **Böhringer et al., 2025 (138); Regenbogen** et al., 2024 (21); **Prost** et al., 2024 (68); **Kalbas** et al., 2024 (174); **Wan** et al., 2023 (58); **Ganeshan** et al., 2023 (1); **Kramer** et al., 2023 (46); **Lodde** et al., 2023 (1); **Takaesu** et al., 2022 (41); **Kress** et al., 2022 (28); **Boudessa** et al., 2022 (127); **Balling**, 2021 (0); **Passias** et al., 2021 (134)	1505 (0)	Simple, safe, minimally invasive and precise method with good clinical results in terms of rapid recovery with early mobilization of patients to maintain autonomy and reduce mortality	20	II	**Sola** et al., 2025; **Klingebiel** et al., 2025; **Mizutani** et al., 2025; **Prost** et al., 2024; **Kalbas** et al., 2024; **Boudessa** et al., 2022; **Passias** et al., 2021	Significant reduction in fluoroscopy time = lower exposure for medical personnel, mean operation time lower, higher accuracy in screw placement, substantial pain relief, enables early mobilization, and demonstrates a low complication rate at 3 months, especially applicable in certain difficult imaging situations, such as morbid obesity, bowel gas interference, and overlapping pelvic structures that make the sacral corridor difficult to discern with traditional 2D fluoroscopy, significant enhancement in screw placement precision, avoiding cortical perforations	Requires access to a hybrid operating room, Longer preparation, including draping and matching 3D scan; Radiation exposure for patients during 3D-navigation for percutaneous SI screws is increased, achieving optimal angulation in the axial plane remains challenging, even with 3D
**Robot**	**Pai** et al., 2025 (2); **Haveman** et al., 2025 (0); **Tian** et al., 2025 (22); **Zhao** et al., 2024 (15); **Vennitti** et al., 2024 (1); **Smith** et al., 2024 (1); **Xing** et al., 2024 (46); **Tian** et al., 2023 (48); **Hao** et al., 2023 (32); **Li** et al., 2023 (57); **Carlson** et al., 2022 (1)	223 (2)	RARIF	11	III	**Haveman** et al., 2025; **Smith** et al., 2024; **Xing** et al., 2024; **Hao** et al., 2023; **Li** et al., 2023	Less operation time, less intraop bleeding and fluoroscopy, higher healing rate, plan and execute screw paths through the narrow bony corridors, chance of skiving is minimized, excellent or good reductions, 6-month follow-up revealed an average modified Majeed score of 81.4, with 85.7%, superiority in accuracy is even more significant in cases of pelvic dysmorphism/higher BMI, fewer guidewire attempts, better ergonomics for the surgeon	Controversial evidence regarding the impact on operative time as some studies have reported increased operative time, whereas others demonstrated reduction with the use of robotics, higher costs
**3D printed templates**	**Wan** et al., 2023 (58)	58 (0)	Special internal template, based on 3D reconstructed model of patient and reverse engineering, provided an alternative method for the placement of iliosacral screw	1	III	**Wan** et al., 2023	Reducing the number of intra-operative fluoroscopies, operation time, and blood loss	Prolonging the patient’s preoperative preparation time (plan the screw channel, perform 3D printing, and finally sterilize the template), once the template was printed, the screw channel was unchangeable
**Cone beam CT with augmented fluoroscopy (CBCT-AF)**	**Sag** et al., 2022 (12)	12 (0)	FDA-approved commercially-available software allows for cone beam CT with augmented fluoroscopy (CBCT-AF) including operator-defined angle-of-attack vectors and warning-line contours that project in 3D on the fluoroscopic image (augmented reality/augmented fluoroscopy)	1	III	0	Augmented fluoroscopy may enable operators without access to CT machines to perform sacroplasty with a greater degree of efficiency and safety	-
**Hyper-inlet view**	**Gosselin** et al., 2022 (34)	34 (0)	Uses additional cranial tilt relative to the traditional inlet view	1	IV	0	Helpful in delineating the spinal canal more clearly and defining the posterior limit of the osseous fixation pathway of the upper sacral segments more precisely	-

**Table 3:** Biomechanical studies (Sai = sacral-alar-iliac)

Studies (Author, Year, Number of included artificial pelvises/cadaver)	Characteristics
**Zaho** et al., 2025 (1)	Bilateral S2AI annular fixation and S1 transsacral screw (BS2AI-S1) offer the best stability in Denis type II fractures
**Ma** et al., 2025 (20)	The percutaneous retrograde sacral alar-iliac screw (RSAIS) for sacroiliac joints with low bone density is superior to sacroiliac screw (SIS) and transsacral-transiliac screw (TSTIS)
**Graul** et al., 2025 (5)	Sufficient stability could be achieved using triangular stabilization using two obliques and an additional transiliosacral screw in a simulated instable sacral bilateral fracture. Cement augmentation did not increase stability.
**Lodde** et al., 2025 (1)	The use of bilateral SI screw or transsacral screw for fixation of unilateral FFP is biomechanically favorable compared to the use of a unilateral SI screw
**Voort** et al., 2024 (5)	In U-type sacral fractures, a modified triangular stabilization construct with S1 pedicle screws improves stability
**Sevillano-Perez** et al., 2024 (20)	When proximal fixation is only up to L5 (vs. up to L4), better stability can be obtained in spinopelvic dissociation
**Du** et al., 2024 (16)	The stability of S1AIS and S2AIS is similar. Both are superior to SIS and TSTIS.
**Grüneweller** et al., 2023 (16)	Using the TriFix-system, implant anchorage and primary stability can be improved in the treatment of iliosacral instability compared to SI-plate
**Cintean** et al., 2023 (10)	Fracture stability was greater and interfragmentary movement was significantly reduced using TITS compared to SI
**Berk** et al., 2023 (24)	Additional cerclage augmentation of S1–S2 transsacral screw fixations may be performed in cases of poor bone quality
**Gonçalves** et al., 2023 (1)	In vertically unstable pelvic ring injuries, using two transiliac screws in S1 is biomechanically superior to the other tested options
**Wu** et al., 2023 (13)	Oblique sacroiliac screws (OSS) in S1 combined with TTS in S2 had the best stability in patients with sacrum dysplasia
**Bradley** et al., 2022 (16)	In type C pelvic ring disruption a novel interdigitating washer design may be superior to using screw constructs
**Carlson** et al., 2022 (1)	Feasibility study: The Mazor X Stealth Edition robot can be used to place screws into the LC-II and trans-ilio-transsacral screw pathways
**Lodde** et al., 2021 (21)	Cement augmentation of one SI screw resulted in significantly less displacement compared to one or two SI screws
**Zheng** et al., 2021 (100)	Iliosacral triangular osteosynthesis can be used in the treatment of posterior pelvic ring injuries, although biomechanical stability is slightly lower than in TTS
**Suero** et al., 2021 (10)	Treatment with non-augmented double-screws provides similar biomechanical stability compared to a single cement-augmented cannulated sacroiliac screw
**Peng** et al., 2021 (1)	Biomechanical analysis of “H”- and “U”-type sacral fractures: bilateral triangular fixation was the strongest fixation, transsacral-transiliac screw yielded great symmetry
**Gierig** et al., 2021 (1)	Use of an additional cross connector in Denis zone 1 sacral fracture reduced the maximum stresses in the fracture area significantly without altering ROM in L4/L5 or L5/sacrum
**Lodde** et al., 2021 (50)	S1/S2 ala-ilium screws were more stable compared to SI screw or SI screw and external fixator in the treatment of FFP IIc fractures
**Zderic** et al., 2021 (27)	The new screw-in screw implant provides higher stability than an SI screw in the treatment of fragility sacrum fractures
**Shannon** et al., 2021 (20)	Treatment of vertically unstable transforaminal sacral fractures with fully threaded transiliac-transsacral fixation may be mechanically superior to partially threaded fixation

The results of these studies have been summarized into themes directly related to the use of percutaneous screw fixation of geriatric posterior pelvic ring lesions. These are as follows:

- i. Use of Navigation and Robotics for screw Placement ii. Use of Screw Augmentation iii. Fixation Methods iv. Novel Implants/Technologies v. Biomechanical studies

i. **Use of navigation and robotics for screw placement**

In the herein study, 20 articles [23, 27, 43–60] assessed conventional 3D computer-assisted navigation, whereas 11 articles [37, 38, 47, 61–68] examined robotic arm–assisted navigation in the management of posterior pelvic injuries (Table 2).

One of the included studies was a meta-analysis by Haveman et al., which evaluated 19 studies comparing conventional 2D fluoroscopy with various navigation techniques for percutaneous sacroiliac screw fixation [47]. The authors demonstrated significantly higher screw placement accuracy (92% vs. 82%) and reduced fluoroscopy time and frequency with navigated techniques, while complication rates were low in both groups. More advanced modalities such as 3D CT and robotic navigation appeared to perform particularly well.

Sola et al. compared in a retrospective study traditional 2D fluoroscopy (*n* = 18) with 3D computer-assisted navigation (*n* = 18) regarding radiation exposure and operation time in patients with fragility fractures undergoing treatment with percutaneous SI screws [43]. The navigated group showed a significant reduction in fluoroscopy and mean operative time with no screw-related complications. In line with these findings, Prost et al. and Passias et al. found that the use of navigation may lead to a significant reduced duration of surgery and a reduced exposure to radiation for both patient and surgeon [50, 59].

Analyzing bone healing, Klingebiel et al. found no significant differences in non-union rates in a matched-pair analysis (navigated versus conventional sacroiliac screw fixation) of 208 patients [44].

Gilli et al. presented the largest case series to date on sacroiliac (SI) screw placement using intraoperative 3D navigation with an O-arm system (241 screws in 158 patients) [23]. This retrospective analysis demonstrated a high level of accuracy: 83.2% of screws were completely intraosseous, and 99.2% showed less than 2 mm of cortical perforation.

Mizutani et al. [45] compared the effectiveness of O-arm-based 3D navigation with conventional fluoroscopy for percutaneous screw fixation in patients with fragility fractures of the pelvis (FFPs). Seventy-two patients were included (O-arm group: 14; fluoroscopy group: 58), with outcomes assessed in terms of screw-placement accuracy, radiation exposure, and surgical parameters. The O-arm group demonstrated significantly lower screw malposition rates (5.7% vs. 20%) and radiation exposure to the surgeon (0.1–0.2 µSv vs. 109.8 mGy), with no reoperations required compared to three in the fluoroscopy group. However, the O-arm group had slightly longer surgical times and higher intraoperative blood loss.

In a retrospective observational study of 53 patients, Mantilla-Mayans et al. demonstrated that 3D-navigated transsacral screw fixation significantly reduces pain and enables early mobilization [46].

Another promising approach in the percutaneous treatment of FFPs is robot-assisted surgery. In a single-center case series from Beijing, Zhao et al. reported 15 patients with displaced FFPs who underwent surgery using an advanced robot-assisted fracture reduction system [62]. Using Matta criteria, they found a 100% success rate evaluating the fracture reduction in postoperative radiographs.

Xing et al. analyzed 46 patients with FFPs who had been treated with percutaneous screw fixation using either TiRobot assistance (*n* = 24) or conventional freehand placement (*n* = 22) of the screws [65]. Of interest, they found a more accurate screw placement, better fracture reduction and early pain relief in the TiRobot-assisted group.

Tian et al. treated Type IV fragility fractures of the pelvis (FFPs) with robotic assisted triangular fixation and reported very good results [38]. However, large-scale studies evaluating this technology are currently lacking, and further evidence is needed to support its use in the management of fragility fractures.

ii. **Use of screw augmentation/sacroplasty**

**Fig. 2:**
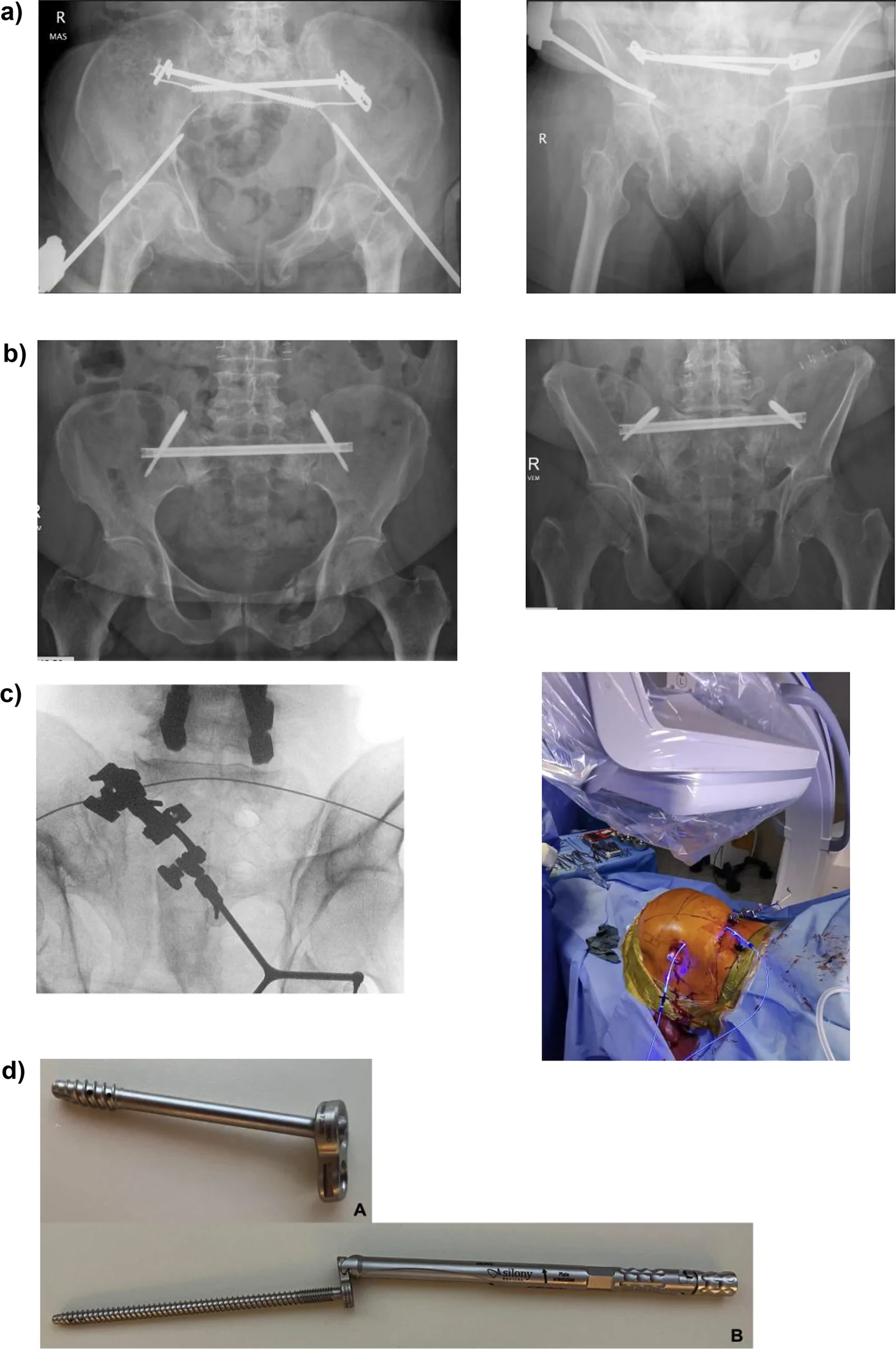

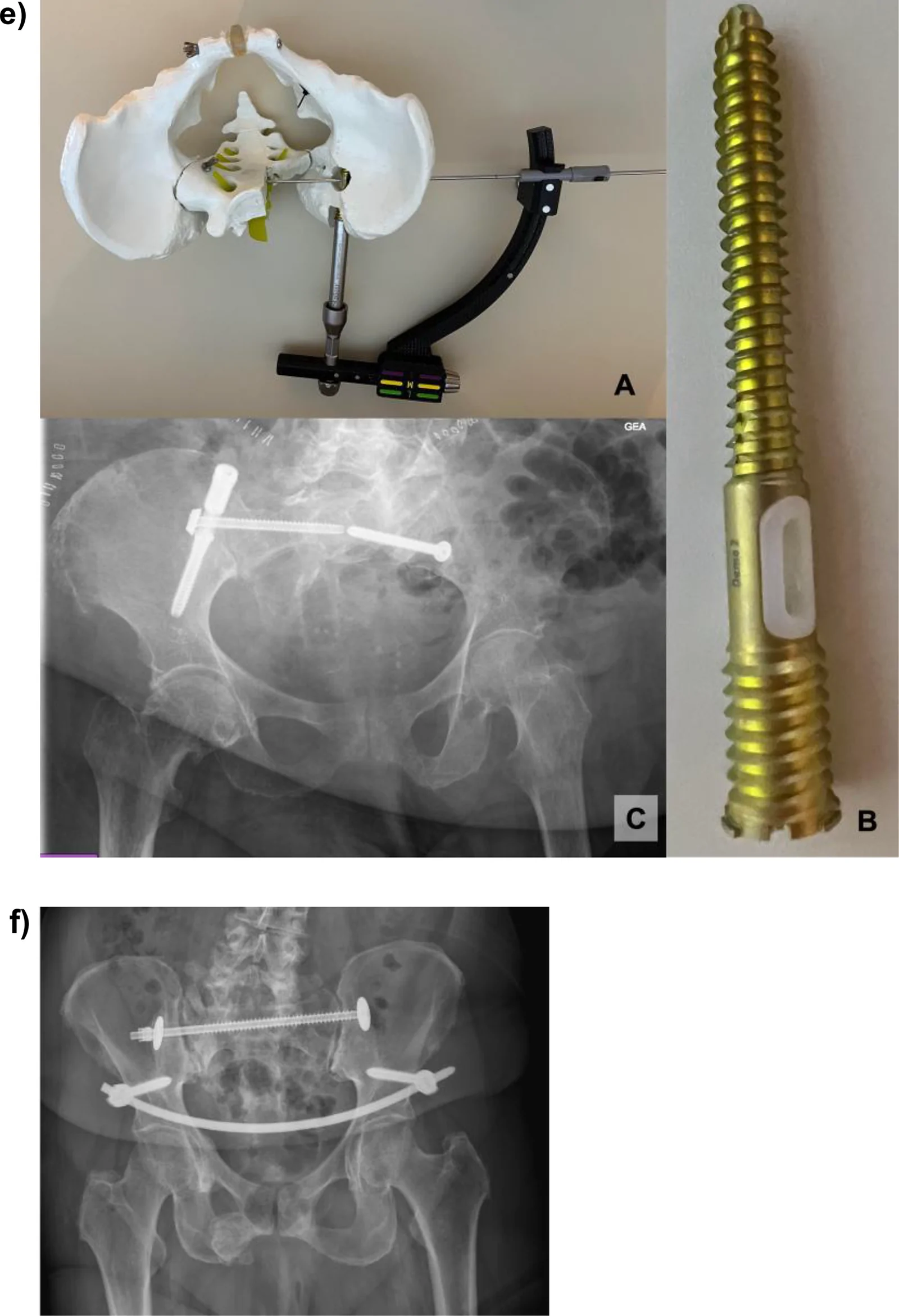
Novel techniques/implants. **a**)Transsacral screws augmented with cerclage wires. Inlet view (left) and outlet view (right). **b**)SACRONAIL® (signus, Alzenau, Germany). The bilateral locked iliac screws add stability to the anterior pelvic ring. A.P. view (left) and outlet view (right). **c**)Photodynamic nails are inserted over a flexible guidewire (left). The balloon polymer is then hardened using blue light (right). **d**) locking plate augmented SI screw (SI-Plate, Silony Medical GmbH, Frauenfeld, Switzerland). A partially threaded (**A**) or a fully threaded/dual-threaded iliosacral screw (**B**) can be used. For the pre-mounted plate that functions as a washer, a short locking screw can be placed into the ilium using a sleeve. **e**)The iliac screw augmented SI screw (TriFix system, Silony Medical GmbH, Frauenfeld, Switzerland). With the use of aiming devices, minimally invasive stabilization of both the anterior and posterior pelvic ring is intended (**A**). The iliac screw is fenestrated to allow the placement of the SI screw (**B**). **f**) ISG rod (axomed, Freiburg, Germany). The anterior pelvic ring is fixed by internal fixation (INFIX)

In the present study, 23 papers analyzed the use of different screw augmentation techniques or sacroplasty [27, 54, 56, 69–86]. Berk et al. augmented transsacral screws with cerclage wires in a biomechanical study to find that **cerclage augmentation** enhances the mechanical stability of the construct [69]. Although this method was applied clinically at our institution (Fig. 2a), it has since been abandoned due to its time-consuming and labor-intensive nature.

**Cement augmentation** of screws is a significantly more popular technique among surgeons; however, it carries the inherent risk of cement extravasation. A large retrospective single-center study by Hartensuer et al. analyzed 448 patients who underwent percutaneous sacroiliac (SI) screw fixation over a 15-year period, totaling 642 inserted screws [83]. Cement augmentation was used in 26% of cases. Cement-augmented FFP patients showed a 25% reduced stay in hospital and a reduced complication risk. Cement-associated complications were seen in 22% without correlation to neurologic impairment. A limitation of this study is that pelvic fractures in younger patients were not excluded, which may have influenced the results.

A recent retrospective study compared two different cement augmentation techniques. One technique involved inserting cannulated screws which are perforated at the tip only (insertion of cement after cement application), while the other used **fenestrated screws** allowing post-insertion cement application [76]. No significant differences were found between the techniques regarding clinical outcomes or safety (cement leakage).

Graul et al. compared in a recent biomechanical study the cemented with the conventional triangular SI fixation of the sacrum for FFP IV fractures. Contrary to the common definition of triangular fixation (spinopelvic fixation and SI screw), here the authors define triangular as 2 oblique screws in the S1 vertebra and 1 transsacral screw [70]. Five osteoporotic cadaver pelvises with standardized bilateral sacral fractures have been tested to show no statistically significant increase of the stability of the construct with cement.

Sacroplasty was the focus of 9 studies [71, 73, 74, 77–79, 81, 82, 86]. The included studies consistently reported significant pain reduction as well as improvements in functional mobility and quality of life following this minimally invasive procedure.

iii. **Fixation methods**

Between 2020 and 2025, 19 studies focusing on different fixation methods were identified. Of these, 14 articles analyzed lumbopelvic/triangular fixation [27–35, 39–42, 70], 2 articles focused on transilial internal fixation [87, 88] and 6 articles analyzed transsacral fixation using ISG-Rod system or transsacral bar [30, 49, 89–92]

- *Lumbopelvic/triangular fixation*

Triangular fixation stabilizes the posterior pelvic ring by combining iliosacral screw fixation with lumbopelvic fixation, typically using pedicle screws placed in L4 or L5 connected to iliac screws to create a triangular construct. This configuration provides strong multiplanar stability, especially for vertically unstable sacral fractures.

Mendel et al. performed a retrospective matched-pair analysis comparing bisegmental transsacral fixation with iliosacral screws with minimally invasive spinopelvic fixation in patients with FFP IVb fractures [30]. Although fluoroscopy time did not differ between these procedures, transsacral fixation showed a significantly lower cut-seam time and was superior to spinopelvic fixation with respect to mobility level attained at discharge.

Gewiess et al. compared the outcome of 52 patients undergoing transiliac-transsacral screw fixation and 36 patients undergoing lumbopelvic fixation with nondisplaced or minimally displaced H-/U-type sacral fragility fractures [33]. Treatment with transiliac-transsacral screw fixation resulted in lower intraoperative blood loss and less frequent radiculopathy than lumbopelvic fixation. However, there were no differences in terms of reducing low back pain or restoring mobility.

In a retrospective analysis of 13 female patients with sacral fragility fractures (mean age: 83.92 ± 6.27 years), Obid et al. [34] demonstrated that an early mobilization following minimally invasive lumbopelvic stabilization is feasible in geriatric patients, as revealed by the Timed Up and Go Test (TUGT) and by the Tinetti Mobility Test (TMT).

b) *Transilial internal fixation*

Transilial internal fixation (TIFI) is a minimally invasive technique in which two pedicle screws are inserted from anatomical entry points medial and caudal to the PSIS on each ilium and connected subfascially with a transverse rod [88]. The method allows closed reduction maneuvers using iliac screws as joysticks and can also be applied safely in patients with a dysmorphic sacrum. Disadvantages include its limited ability to provide compression compared with iliosacral screws and a higher risk of wound-related complications. According to the current literature, the technique has not been evaluated in fragility fractures, as all reported cases involved high-energy injuries [87, 88].

c) *Transsacral fixation*

Pelvic fragility fractures are more and more seen as a progressive disease. A unilateral vertical sacral lesion may, over time, be followed by a contralateral lesion and eventually a connecting transverse fracture [93]. The use of bilateral sacroiliac or transsacral screw fixation can help offload the contralateral, initially uninjured side, thereby reducing mechanical stress and potentially lowering the risk of adjacent fracture development or progression [7, 94].

The 7.5 mm ISG-Rod system (Axomed, Freiburg, Germany) had its market launch in 2017 and has been increasingly used to treat fragility fractures since then (Fig. 2f). It consists of a threated transsacral rod secured with washers or nuts on both iliac sides, creating a rigid, bilateral construct. Through this implant design a controlled compression of the fracture is possible and the risk of implant loosening is eliminated. Fischer et al. compared in a retrospective study this implant (*n* = 48) with transsacral partial threaded screws (*n* = 26) to find no significant differences in complication rates [89]. The 6.0 mm sacral bar (Depuy-Synthes, Oberdorf, Switzerland) has also been evaluated in this study. Since the technique of insertion of this implant is not minimal invasive, this has not been included in our analysis. Wagner et al. showed in a retrospective study of 85 patients that transsacral bar osteosynthesis provided low mortality and high mobility for patients with fragility fractures [92].

## Novel Implants/technologies

Twelve articles included to this study focused on novel implants, such as Sacronail [95, 96], CurvaFix [97], Photodynamic nails [98], Gull wing plate [99–101], E. spine tanit [102], SI Plate/TriFix, modified LC II screw [103] or the retrograde sacral alar-iliac screw (RSAIS) [104].

- *Sacronail*

The SACRONAIL® (Signus, Alzenau, Germany) is a newly developed, minimally invasive, transsacral locking system (Fig. 2b) [95]. Advantages of this implant design are the additional rotational stabilization of the pelvis through the bilateral locked iliac screws and the resistance to loosening of the implant. Despite these theoretical advantages and the positive experience of using this implant in our institution, no larger clinical or biomechanical studies are available. Biomechanical testing and comparative studies with transsacral screws are needed.

b) *CurvaFix*

CurvaFix ® (Bellevue, Washington, USA) is a novel device that is flexible during insertion. Once in place, it locks into a rigid, curved shape that resists rotation and loosening, providing stable fixation across complex pelvic anatomies, including dysmorphic S1 corridors or areas with pre-existing hardware [97]. No big clinical or biomechanical studies are yet available.

c) *Photodynamic nails (PDNs)*

Photodynamic Bone Stabilization Systems have been used a lot for treatment of pathological fracture of the extremities (Fig. 2c). A flexible balloon catheter is inserted in the bone and filled via syringe with a liquid polymer that hardens upon exposure to light. Clunk et al. used this technique in 14 patients to stabilize impending pathologic and insufficiency fractures of the sacrum in oncologic patients with poor bone quality [98]. In our institute we have used this technique also to treat not tumor associated fragility fractures of dysmorphic sacrum, with good results.

d) *Gull wing plate*

The gull wing plate (GWP; OMIC Corporation, Japan) is a minimally invasive posterior pelvic fixation technique in which a pre-contoured locking plate is passed subfascially through two small incisions, rotated into a prepared iliac groove, and secured with long cancellous screws toward the AIIS and tricortical locking screws across the iliosacral joint [99]. According to the current literature article, the technique has not been evaluated in fragility fractures, as all reported cases involved high-energy injuries.

e) *E. Spine tanit*

E. Spine Tanit (Euros, France) is a minimally invasive system that connects iliosacral screws with bilateral iliac screws. The technique has been designed specifically for fragility fractures. Sawada et al. describe in a case report its application in a 90-year-old patient with an FFP IVb fragility fracture of the pelvis [102].

f) *SI Plate/*triFix

A recent interesting biomechanical study compared two novel implants developed for the treatment of fragility pelvic fractures: the **locking plate augmented SI screw** (SI-Plate, Silony Medical GmbH, Frauenfeld, Switzerland) with the **iliac screw augmented SI screw** (TriFix system, Silony Medical GmbH, Frauenfeld, Switzerland). While the SI-Plate, consists of the double-threaded iliosacral screw with a pre-mounted plate that acts as a washer and provides the option of placing a short locking screw in the ilium (Fig. 2d), the TriFix system consists of a cannulated iliac screw though which an Si screw is placed (Fig. 2e) [105]. Both implants demonstrated comparable initial stiffness, but the TriFix showed greater durability under physiological loading.

g) *Modified LC II screw*

Deng et al. describe a modified LC-II screw fixation for treating FFP IIIa and IIIb fragility fractures [103]. Compared to the conventional LC-II screw, the entry point in this modified technique is more lateral and enters the sacroiliac joint through the iliac osseous corridor at an angle of 5°–10° to the osseous corridor of the LC-II screw. Using this technique, the path of the osseous corridor is shorter, as it does not pass through the curved bone surface of the iliac fossa. This reduces the risk of the ‘in–out–in’ phenomenon. Operative time was significantly reduced and preliminary results show a satisfactory clinical outcome [103].

h) *Retrograde sacral alar-iliac screw (RSAIS)*

As the spinous process can block the percutaneous implantation of a conventional SAIS, Ma et al. [104] describe a retrograde sacral alar-iliac screw for fixation of the sacroiliac joint. Biomechanical testing revealed that RSAIS fixation stability for sacroiliac joints with low bone density is superior to classical sacroiliac screw and transsacral-transiliac screw [104]. This new technique was also applied to five patients (four males, one female). With these preliminary results, Ma et al. could demonstrate that RSAIS placement is simple, safe and provides good clinical outcomes, as measured by functional recovery (e.g. Majeed scores).

## Biomechanical studies

Twenty-two biomechanical studies were included in this scoping review [28, 31, 36, 40, 68–70, 80, 85, 104–115]. As treatment with percutaneous sacroiliac screw fixation in fragility sacrum fractures can result in high rates of turn-out, Zderic et al. [115] presented a new screw-in-screw prototype: it consists of a standard 7.3 mm cannulated SI-screw adopted to accommodate a 2.7 mm antirotation locking head screw at a 15° set angle. After biomechanical testing in 27 artificial pelvises, the authors concluded that the new screw-in-screw implant provides higher stability than SI screws.

Cintean et al. compared transiliac-transsacral and sacroiliac screw osteosynthesis in 10 osteoporotic cadaver pelvises with FFP IIc fractures [107]. The fractured side was then loaded in a one-leg stance test setup. A significantly higher stability for gap angle, flexion, vertical movement and overall stability was found in the transiliac-transsacral group.

Wu et al. used finite element analysis (FEA) to investigate the biomechanical performance of oblique sacroiliacal screws (OSS) combined with transiliac-transsacral screws (TTS) for pelvic fracture or sacroiliac dislocation in patients with dysplastic sacrum [109]. They found that the combination of OSS in S1 and TSS in S2 had the best biomechanical stability.

Lodde et al. simulated 50 FFP type IIc fractures in artificial pelvises to compare different fixation techniques including SI screw, SI screw plus supra-acetabular external fixator, SI screw plus plate, SI screw plus retrograde transpubic screw, or S1/S2 ala-ilium screws [111]. Although open reduction and internal fixation with a plate provided the highest stability, percutaneous fixation using S1/S2 ala-ilium screws is a successful alternative.

## Discussion

With an increasingly aging population and an increasing incidence of osteoporotic fractures we will encounter more frequently pelvic ring fractures. This review has aimed to provide an update on studies published over the past 5 years. Surgical treatment of said fractures remains problematic due to bone quality (fragility secondary to osteoporosis), patient co-morbidities and anatomical variations of the bone corridors used for stabilization of fractures. Despite the advances made in theatre equipment, imaging modalities and medical treatment of comorbidities, stabilization of these fractures remains challenging.

### Use of navigation for screw placement

According to recent literature, the use of 3D computer-assisted navigation or O-arm technology for iliosacral screw placement significantly increases accuracy and reduces complications such as cortical perforation [44]. This technology offers substantial advantages over 2D fluoroscopy, particularly in patients with morbid obesity, in the presence of bowel gas, or when overlapping pelvic structures obscure anatomical landmarks. Additionally, patient and surgeon radiation exposure and operative time are significantly reduced in comparison to conventional intraoperative 2D imaging [9, 10]. However, the use of navigation depends on the hospital’s structural requirements. Navigation requires access to an operating room with 3D fluoroscopy and longer preparation time, including draping and matching the 3D scan. Despite the aforementioned advantages, achieving optimal angulation in the axial plane remains challenging, even with 3D technology. As demonstrated by Mantilla-Mayans et al., navigated transsacral screw placement significantly reduces patient pain and enables early mobilisation [46].

At our orthopaedic trauma centre, we rely on navigation in all cases involving a dysmorphic sacrum, narrow osseous corridors, obese patients, or planned cement augmentation—representing more than 70% of cases. With improving technology and growing team experience, operative time is no longer significantly prolonged compared with standard 2D fluoroscopy. We believe, however, that the ability to perform this operation without navigation remains essential—not only to avoid dependence on the technology, but also to verify implant placement if navigation fails, for example due to a loose reference marker. Nevertheless, if intraoperative navigation is not available, Gosselin et al. describe the hyperinlet view, which uses additional cranial tilt relative to the traditional inlet view, to supplement the intraoperative inlet and outlet views [116]. This procedure has proven helpful in better delineating the spinal canal and thereby better defining the posterior limit of the osseous fixation pathway of the upper sacral segments.

### Implant loosening: Use of cement?

The incidence of screw loosening after iliosacral screw fixation for FFPs has been reported to be high (between 10 and 20%) [7]. Using bone cement to augment the density of the (osteoporotic) host bone can be very helpful in the treatment of FFPs. Compared to nonaugmented screws, cement augmentation increases anchorage and reduces displacement, as well as significantly reducing cut-out distance [72]. Cement augmentation of fenestrated SI screws can mitigate this risk; however, cement extravasation into the neural foramina or spinal canal remains a feared complication. A precise screw corridor is therefore imperative.

Both sacroplasty and screw augmentation can significantly alleviate patients’ pain [74]. When using these cement augmentation techniques, the potential risks of cement leaking into the fracture gap or spinal canal, and of cement embolisms, must be taken into account. Nevertheless, these techniques are safe procedures with a low rate of complications if performed properly [74]. At our institute, we have very limited experience with sacroplasty, as it does not provide mechanical stability to the pelvic ring and the fracture may continue to displace. Cement augmentation is also used only rarely, because transsacral–transiliac screws generally achieve excellent purchase even in osteoporotic bone.

Cerclage reinforcement can also be applied to avoid loosening of the screws. Biomechanical studies have shown that augmenting S1–S2 transsacral screw fixation with cerclage can result in significantly less combined angular intersegmental movement [12]. This technique is no longer employed at our clinic because it is time-consuming and labour-intensive, and clinical evidence remains limited. In the short term, cerclage may be more cost-effective than cement augmentation; however, when potential complications and revision rates are considered, cement augmentation may prove more economical in the long term. Robust cost-effectiveness studies are, however, lacking.

### Spinopelvic dissociation: Are transsacral screws a safe treatment option?

**Fig. 3:**
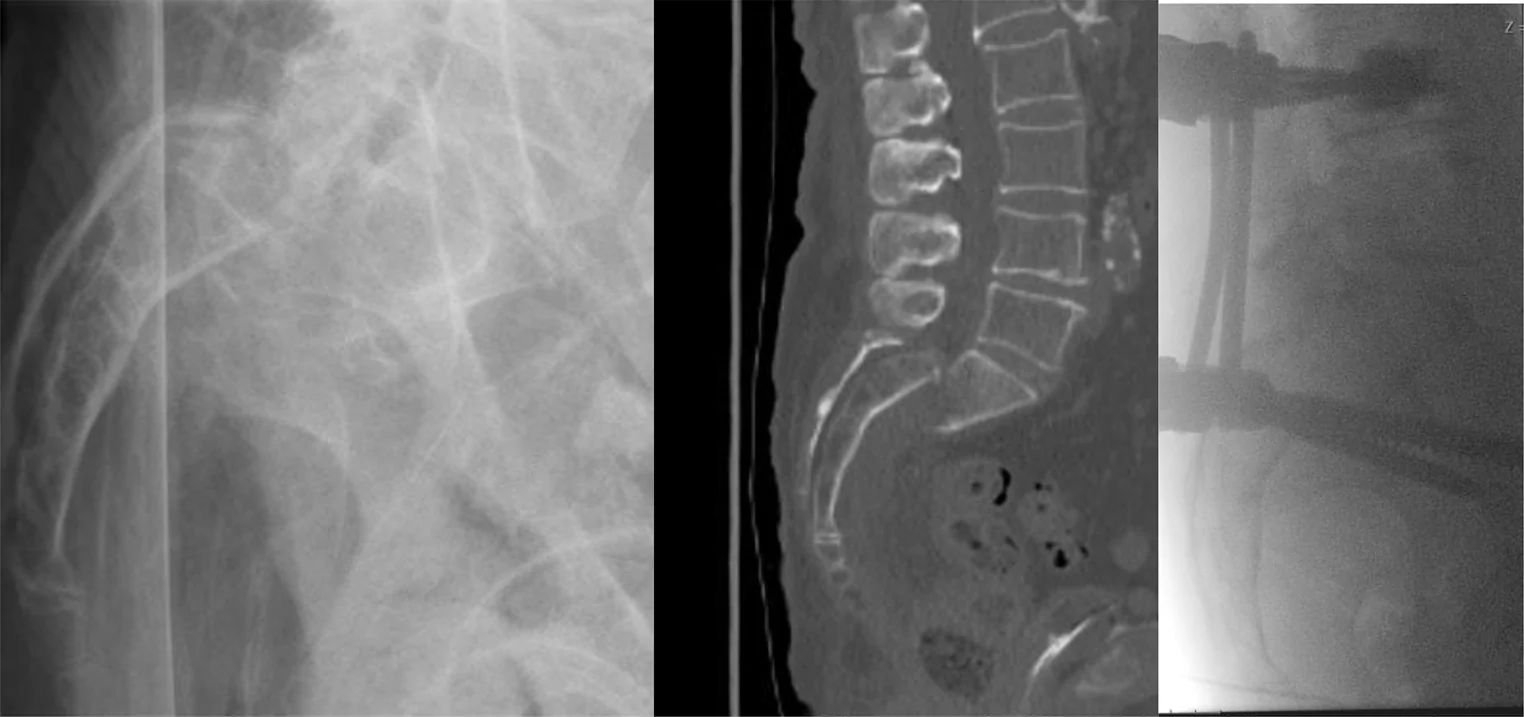
87-year-old female patient with a displaced FFP IVb fracture and spinopelvic dissociation, without neurological impairment. Reduction and fixation using minimally invasive lumbopelvic fixation (right figure)

Spinopelvic dissociation injuries occur in 21% of patients with fragility fractures of the pelvis (Fig. 3) [2]. Although these injuries are typically treated with lumbopelvic fixation, patients with undisplaced or minimally displaced U/H-type sacral fragility fractures can undergo surgery using bisegmental transsacral-transiliac screw fixation. Mendel et al. found that bisegmental transsacral stabilization produced a better subjective outcome than lumbopelvic stabilization in cases of bilateral fragility fractures of the sacrum [29]. Additionally, transsacral-transiliac screw fixation resulted in less blood loss and shorter operating times than lumbopelvic fixation [33]. In our experience it is critical to analyze the fracture pattern in CT since meaningful transsacral screw corridors with a corridor width ≥ 8 mm and no transverse fracture at the screw level are present in only 80% of cases [117]. In addition, the transverse component of the fracture is located in S2 in 42% of the fractures. In our clinical practice, spinopelvic fixations are used in approximately 25% of the surgically treated IVb fractures (H/U fractures)

### Novel implants

The SACRONAIL®, as a transsacral locking system has been used more than 20 times in our clinic and produced excellent results. However, scientific evidence for this implant is scarce. In a prospective pilot study, Marintchev et al. confirm our experiences finding that the SACRONAIL® contributed to a significant improvement in function and well-being in patients with posterior pelvic ring fractures [96]. The authors suggest that these favorable outcomes may be attributable to the additional stabilization of the anterior pelvic ring provided by the iliac screws. The concept of reinforcing the anterior pelvic ring through a posterior construct is also employed in TriFix and E. Spine Tanit, where SI screws are connected to iliac screws to achieve this additional stabilization.

Regarding fracture compression, ISG-rod systems are gaining popularity in Germany. However, despite their long-standing availability on the market, robust scientific evidence supporting these fixation systems is still lacking.

As far as curved implants are concerned, photodynamic nails (IlluminOss, IlluminOss Medical, Inc., Rhode Island, USA) have been used in our clinic not only for metastatic lesions but also for the treatment of fragility fractures. The customizable nature of these implants allows them to be adapted to individual pelvic anatomy and, therefore, could potentially reduce the risk of mechanical failure [24]. The procedure is more time-consuming than SI screw fixation, as polymer hardening can take up to 10 minutes. Infections are exceedingly rare, likely due to the infrared light used to cure the polymer. Implant removal is challenging but feasible and has been successfully performed in our department. Although the technique does not allow for fracture compression, postoperative pain relief is typically immediate. However, clinical studies are still needed to validate its effectiveness for this indication.

### Advantages and limitations of the study

This scoping review provides an overview of the latest advances and current evidence for percutaneous stabilization of the posterior pelvic ring, based on a review of literature from the last five years. The systematic search was performed in PubMed and Cochrane Library. Other major databases such as Embase or Scopus were not included, which is a limitation of the present study. As FFPs are becoming increasingly relevant in an ageing society, this knowledge is becoming ever more important for trauma surgeons. However, as some of the new implants and technologies have only been described in a small number of cases so far, few studies involving patients under the age of 65 were also included in this study [95, 96, 99]. Many new techniques and implants show promising results. This review’s findings should be interpreted with caution due to the lack of high level of evidence in many of the analyzed studies including case reports. Despite this some recommendations could be made based on the available evidence which must be further supported with further studies in the future.

### Future directions of work

Future research should focus on prospective, randomized, comparative (with SI screws as the golden standard) trials of these evolving techniques. The objective is to optimize patient selection, minimize adverse events and improve surgical strategies based on scientific evidence.

## Conclusion

A lot of new implants (e.g. CurvaFix®, ISG-Rod, SI-Plate, TriFix, SACRONAIL®), techniques to augment conventional SI screws (cement, cerclage) and technologies (3D computer-assisted navigation/O-arm/Robot) have been described and were introduced in clinical practice in the last five years. While further comparative studies and evidence is desirable, the most important conclusions of this study are the following:

- Percutaneous transsacral techniques are increasingly recognized as effective and safe minimally invasive options for FFP/sacral fractures.
- 3D navigation enhances implant accuracy and may reduce radiation and overall operative time.
- Transsacral bars offer biomechanical superiority and lower loosening rates - but require suitable sacral corridor anatomy.
- Novel implant designs, like the SACRONAIL®, propose enhanced stability; these require further clinical validation.
- Future research should include prospective, randomized comparative trials across these evolving techniques to optimize patient selection, minimize complications, and refine surgical strategies.

## Data Availability

No datasets were generated or analysed during the current study.
